# Back-to-base Versus In-transit Machine Perfusion in Donation After Circulatory Death Liver Transplantation: Insights From a National Registry

**DOI:** 10.1097/TXD.0000000000001925

**Published:** 2026-03-04

**Authors:** Isaac S. Alderete, Qimeng Gao, Nihal Aykun, Mohamed Mustafa Diab, Nader Abraham, Siavash Raigani, Brian Shaw, Aparna Rege, Lisa McElroy, Deepak Vikraman, Kadiyala Ravindra, Bradley H. Collins, Stuart J. Knechtle, Debra L. Sudan, Andrew S. Barbas

**Affiliations:** 1 Department of Surgery, Duke University Medical Center, Durham, NC.

## Abstract

**Background.:**

Normothermic machine perfusion (NMP) improves utilization of extended criteria liver grafts, but the optimal delivery strategy—whether in-transit or back-to-base—remains uncertain.

**Methods.:**

Adult recipients of donation after circulatory death (DCD) liver transplants between January 1, 2022, and January 1, 2024, were identified using the national transplant database. In-transit NMP was defined as grafts coded as machine perfused; back-to-base NMP was inferred for noncoded grafts with a cold ischemia time of ≥10 h. Baseline characteristics, geographic distribution, and outcomes—including acute rejection, length of stay, and graft survival—were compared. Multivariable Cox regression was used to adjust for dialysis and recipient hospitalization status. A sensitivity analysis was performed, limited to cases with cold ischemia time of ≥10 h across both groups.

**Results.:**

Among 1217 DCD liver transplants using NMP, 936 (77%) were in-transit and 281 (23%) were back-to-base. In-transit NMP was more commonly used in the Western United States, whereas back-to-base was concentrated in the Midwest and Southeast. In-transit recipients had higher rates of pretransplant dialysis (3.1% versus 0.7%; *P* < 0.05) and shorter preservation times (14.1 versus 16.0 h; *P* < 0.05). Median hospital stay was shorter in the in-transit group (8 versus 9 d, *P* < 0.001). There were no significant differences in acute rejection (*P* = 0.15) or 1-y graft survival (93.3% versus 90.5%, *P* = 0.23). In adjusted analysis, back-to-base NMP was not associated with increased graft failure risk (hazard ratio 1.46; 95% confidence interval, 0.90-2.38; *P* = 0.13). Findings were consistent in the sensitivity analysis (n = 1001).

**Conclusions.:**

In-transit and back-to-base NMP strategies yield comparable clinical outcomes in DCD liver transplantation. Strategy selection may be guided by logistical infrastructure and center-level expertise without compromising recipient outcomes.

## INTRODUCTION

Liver transplantation remains the definitive treatment for end-stage liver disease, but the demand for suitable donor organs continues to exceed supply.^1^ To address this shortage, transplant programs have increasingly turned to donation after circulatory death (DCD) donors and other extended criteria grafts.^2,3^ However, these organs are more susceptible to ischemic injury and posttransplant complications, leading to higher discard rates and worse early outcomes.^4,5^ Normothermic machine perfusion (NMP) offers a promising solution by enabling physiologic assessment and reconditioning of marginal grafts, with the potential to expand the donor pool, streamline logistics, and improve transplant outcomes.^6-8^

Currently, 2 Food and Drug Administration (FDA)-approved NMP platforms are in clinical use in the United States: the TransMedics Organ Care System (OCS), approved in September 2021, and the OrganOx Metra system, approved in December 2021.^9,10^ Both devices have demonstrated efficacy in clinical trials and real-world practice, with evidence suggesting reductions in ischemia/reperfusion injury, lower discard rates, and improved short-term outcomes.^7,8^ Despite their shared goals, these platforms differ fundamentally in implementation. The OCS Liver is approved for in-transit use, allowing continuous perfusion from donor to recipient hospital. In contrast, the OrganOx Metra is predominantly used in a back-to-base manner, where grafts are first transported on ice to the transplant center and then ultimately perfused before transplant. Although both approaches are now integrated into clinical workflows, no clinical trials or national studies have directly compared these 2 strategies, and it remains unclear which is better suited to specific clinical scenarios.

Accordingly, this study aims to compare back-to-base versus in-transit NMP strategies using national registry data from adult DCD liver transplants performed between 2022 and 2024. We hypothesize that although outcomes between the 2 strategies will be comparable, differences in patient and donor selection may reflect distinct clinical use cases and logistical considerations.

## MATERIALS AND METHODS

### Data Source and Study Population

We conducted a retrospective cohort study using data from the Scientific Registry of Transplant Recipients (SRTR), including adult liver transplants performed through December 30, 2024. Liver transplant recipients aged 18 y or older who received organs from DCD donors between January 1, 2022, and January 1, 2024, were identified in the DECEASED_DONOR and LIVER_DATA files. This study period was chosen to capture contemporary utilization of NMP after FDA approval of both the TransMedics and OrganOx systems^9^ while ensuring adequate 1-y follow-up. We excluded pediatric recipients, multiorgan transplants, retransplants, and cases with missing data on machine perfusion status.

This study was deemed exempt by the Duke University institutional review board as it used publicly available, de-identified registry data.

### Classification of Preservation Strategy

The primary exposure was organ preservation strategy, categorized as either in-transit NMP or back-to-base NMP. Livers coded as machine perfused in the SRTR data set (LI_MACHINE_PERFUSION = “Y”) were classified as in-transit NMP, consistent with use of the TransMedics OCS, which maintains normothermic conditions during transport from the donor hospital to the transplant center. Livers not coded as machine perfused (LI_MACHINE_PERFUSION = “N”) but with a cold ischemia time (CIT) exceeding 10 h were classified as back-to-base NMP, consistent with clinical application of the OrganOx Metra system. This classification was guided by recent registry-based analyses and correspondence highlighting systematic underreporting of machine perfusion in cases where perfusion is initiated after arrival at the transplant center.^10,11^ In the current SRTR schema, machine perfusion status is captured at the donor hospital, resulting in misclassification of back-to-base cases as non–machine perfused. Additionally, clinical experience suggests that DCD livers preserved solely with static cold storage (SCS) are rarely transplanted after prolonged cold times,^12^ further supporting this threshold-based classification.

**FIGURE 1.**
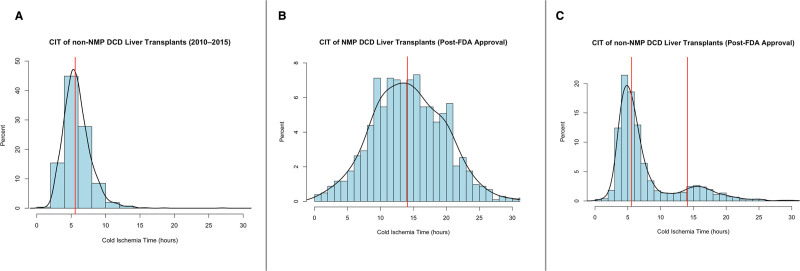
CIT distributions for DCD liver transplants before and after FDA approval of NMP. A, CIT distribution for non-NMP DCD liver transplants performed from 2010 to 2015, representing the pre-FDA approval and preclinical trial era. The vertical red line indicates the median CIT of 5.6 h. B, CIT distribution for DCD liver transplants reported as undergoing NMP from January 2022 to January 2024 (post-FDA approval). The vertical red line represents the median CIT of 14.1 h. C, CIT distribution for DCD liver transplants was not reported as undergoing NMP from January 2022 to January 2024 (post-FDA approval). Two vertical red lines indicate the median CITs from 5.6 h (A) 14.1 h (B), respectively, for reference. CIT was defined according to SRTR conventions, representing the total graft preservation time from aortic cross-clamp to anastomosis. For grafts undergoing machine perfusion, this value may include both cold storage and time on device, as the registry does not distinguish between preservation phases. CIT, cold ischemia time; DCD, donation after circulatory death; FDA, Food and Drug Administration; NMP, normothermic machine perfusion; SRTR, Scientific Registry of Transplant Recipients.

To further validate this classification strategy, we modeled CIT distributions similar to Munoz et al^10^ across 3 distinct cohorts: DCD liver transplants performed in the premachine perfusion era (2010–2015), post-FDA approval DCD grafts coded as machine perfused, and post-FDA approval DCD grafts coded as non–machine perfused. Histograms with kernel density overlays were generated to visualize the CIT distribution in each cohort (Figure 1). The pre-MP era cohort showed a unimodal CIT distribution with a median of approximately 5.6 h and a few cases exceeding 10 h. In contrast, machine-perfused grafts in the post-FDA era demonstrated a rightward shift in distribution, with a median CIT near 14.1 h. Among post-FDA non–machine-perfused grafts, the distribution was bimodal, with a distinct secondary peak at 14–16 h that closely mirrored the machine-perfused cohort. This pattern supports the hypothesis that most of these grafts likely underwent unrecorded back-to-base perfusion. It is important to note that in the SRTR database, CIT reflects total graft preservation time (from cross-clamp to anastomosis). The registry does not separate time spent in cold storage from time on the machine perfusion device.

### Outcomes

The primary outcome was all-cause graft failure at 30 d and 1 y, defined as a composite of recipient death or graft failure necessitating retransplantation. Secondary outcomes included hospital length of stay and episodes of acute rejection during the index hospitalization.

### Statistical Analysis

Baseline characteristics were compared using the Wilcoxon rank-sum test for continuous variables and the chi-square test for categorical variables. Kaplan-Meier survival analysis was used to evaluate graft survival, with comparisons across preservation strategies performed using the log-rank test. To estimate the association between preservation strategy and 1-y composite graft failure (death or retransplant), we initially selected covariates for multivariable modeling based on clinical relevance and prior literature.^13,14^ These included donor age, sex, and diabetes history; recipient age, sex, Model for End-Stage Liver Disease (MELD) at transplant, primary diagnosis, pretransplant dialysis status, and medical condition at transplant; and transplant-related factors including the distance between donor and recipient hospitals.

Given the limited number of events and the risk of overfitting, we applied penalized Cox regression using an elastic net approach (α = 0.5) with 10-fold cross-validation to inform variable selection.^15,16^ A least absolute shrinkage and selection operator-penalized Cox model was fit as a sensitivity analysis to assess robustness. Variables with coefficients shrunk to zero in both models were not retained in a final traditional Cox model. The proportional hazards assumption was assessed using Schoenfeld residuals. Missing data among covariates were minimal (<1%) and were deemed missing at random; therefore, we handled them using mean or mode imputation.^17^

To address potential bias introduced by the CIT-based classification of perfusion strategy, we conducted a sensitivity analysis restricting both groups to recipients with a CIT of ≥10 h. This approach ensured that comparisons between in-transit and back-to-base perfusion strategies were not confounded by differences in preservation time inherent to the classification method. We repeated baseline comparisons and fitted a Cox proportional hazards model using the same covariates as in the primary analysis.

To examine whether the preservation strategy affected how long patients waited for transplant, we compared waitlist time using a mixed-effects linear model. The model included a random effect for transplant center to account for center-level behaviors and adjusted for age, sex, MELD score, and diagnosis as fixed effects. Wait time was log-transformed to normalize the distribution, and results were converted back to days for interpretation. Differences between groups are presented as ratios of average wait times and as adjusted mean days. All analyses were performed using RStudio (version 4.4.3).

## RESULTS

### Study Population and Exposure Classification

**FIGURE 2.**
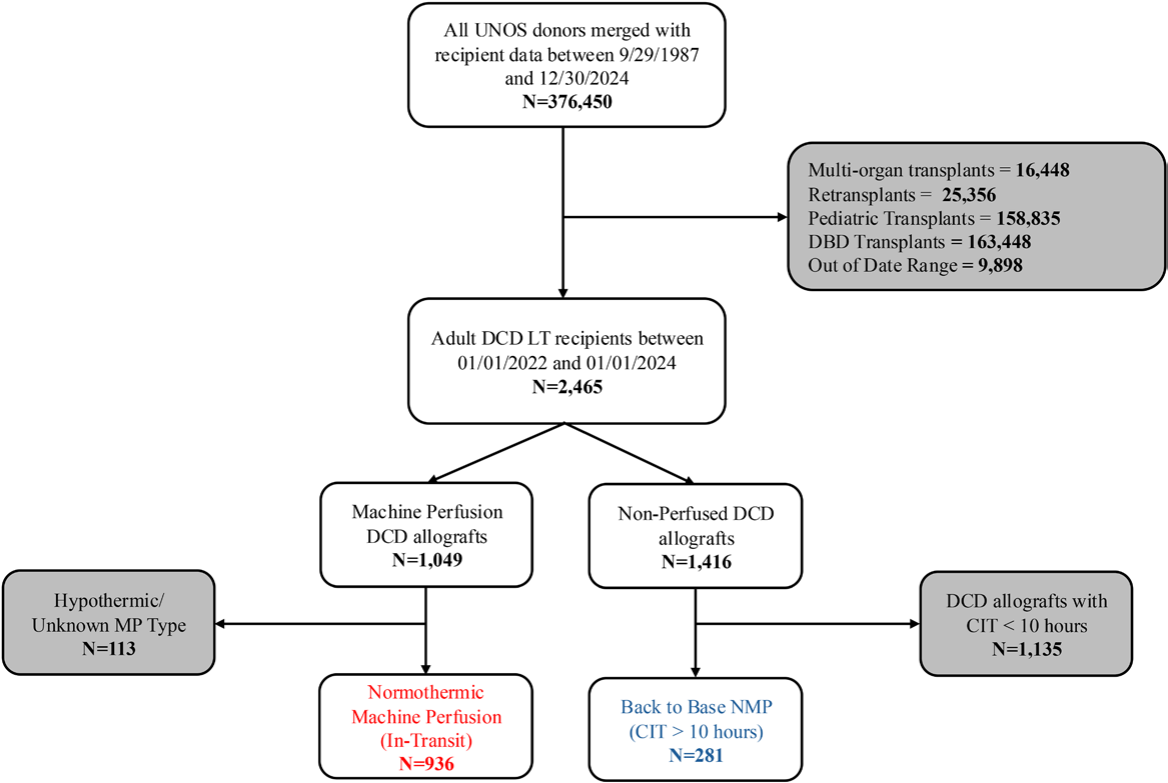
Cohort selection and classification of NMP platforms in adult DCD LT (2022–2024). From the national UNOS registry, we identified 2465 adult recipients of DCD LTs performed between January 1, 2022, and January 1, 2024. Among 1049 grafts reported as having undergone machine perfusion, we excluded 113 that received hypothermic or unknown perfusion types. The remaining 936 were categorized as in-transit NMP allografts, defined as normothermic perfusion performed during transport. Among 1416 nonperfused DCD grafts, 281 were inferred to have undergone back-to-base NMP, defined as grafts with a CIT >10 h—suggesting transport to a perfusion facility before transplantation. The remaining 1135 grafts with CIT < 10 h were excluded from the comparative analysis. CIT, cold ischemia time; DBD, donation after brain death; DCD, donation after circulatory death; LT, liver transplantation; MP, machine perfusion; NMP, normothermic machine perfusion; UNOS, United Network for Organ Sharing.

Following application of exclusion criteria, we identified 2465 adult recipients of DCD liver transplants performed between January 1, 2022, and January 1, 2024. Of these, 936 grafts were classified as undergoing in-transit NMP, and 281 were inferred to have undergone back-to-base NMP. A total of 1248 grafts were excluded: 113 received hypothermic or unknown perfusion types, and 1135 had a CIT of <10 h and were presumed to have undergone SCS (Figure 2). The classification of preservation strategy was supported by the distribution of CIT across historical and contemporary cohorts. Notably, post-FDA DCD grafts not coded as machine perfused exhibited a bimodal CIT distribution, with a secondary peak aligning with machine-perfused grafts, suggesting misclassified back-to-base NMP cases (Figure 1).

### Center-level and Geographic Utilization of Machine Perfusion

**FIGURE 3.**
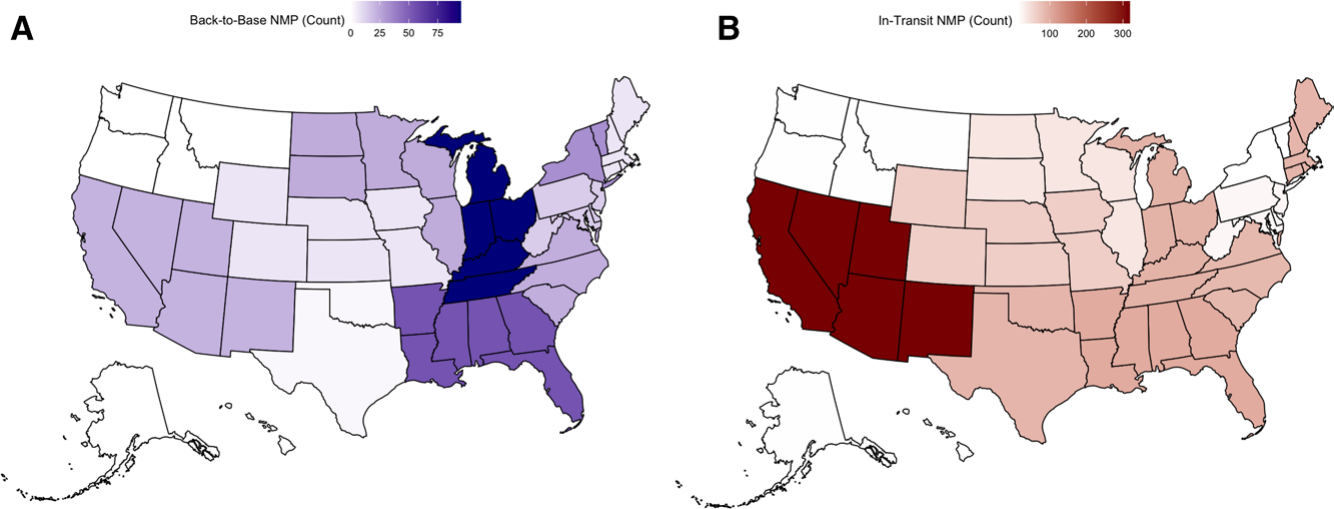
Geographic distribution of back-to-base vs in-transit NMP DCD liver transplants by UNOS Region (2022–2024). Choropleth maps displaying the volume of DCD liver transplants using NMP from 2022 to 2024, stratified by perfusion strategy and shaded by UNOS Region. A, Transplants performed using back-to-base NMP, where perfusion is initiated after cold storage at the transplant center. B, Transplants using in-transit NMP, where perfusion is initiated at the donor hospital and maintained during transport. The highest use of back-to-base NMP occurred in UNOS Region 10 (IN, KY, MI, OH, TN), whereas the highest use of in-transit NMP occurred in UNOS Region 5 (AZ, CA, NV, NM, UT). DCD, donation after circulatory death; NMP, normothermic machine perfusion; UNOS, United Network for Organ Sharing.

**FIGURE 4.**
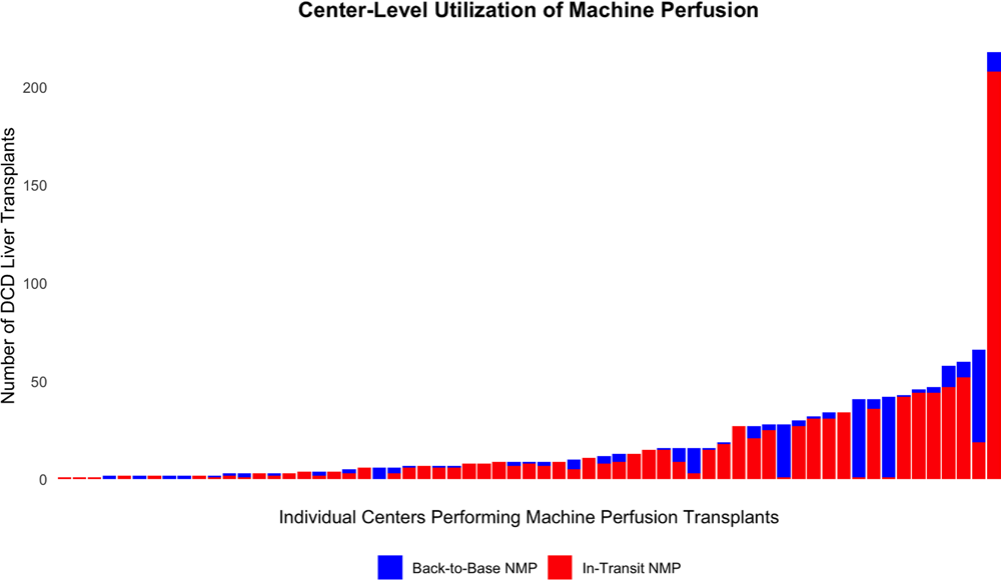
Center-level utilization of machine perfusion for DCD liver transplantation. Number of DCD liver transplants using NMP performed at each transplant center from January 2022 to January 2024. A total of 63 centers performed at least 1 DCD-NMP liver transplant during the study period. Bars represent individual centers and are color-coded by perfusion strategy. DCD, donation after circulatory death; NMP, normothermic machine perfusion.

A total of 63 centers performed at least 1 DCD-NMP liver transplant during the study period. Most centers used both preservation strategies; however, substantial regional and center-level variation was observed. Back-to-base NMP utilization was concentrated in the United Network for Organ Sharing (UNOS) Region 10 (IN, KY, MI, OH, TN), whereas in-transit NMP was most used in UNOS Region 5 (AZ, CA, NV, NM, UT; Figure 3). Center-level analysis demonstrated that a minority of high-volume programs accounted for the majority of in-transit NMP use, whereas back-to-base NMP was more evenly distributed across centers (Figure 4).

### Baseline Characteristics

Baseline recipient, donor, and transplant characteristics are summarized in Table 1. The distributions of recipient age, sex, race/ethnicity, body mass index, MELD score at transplant, and waitlist time were similar across preservation strategies. A difference in admission status was observed, with 4.1% of in-transit recipients and 1.1% of back-to-base recipients listed as intensive care unit (ICU) at the time of transplant (*P* = 0.045). Primary diagnosis differed between groups (*P* = 0.028), with noncholestatic cirrhosis being the most common indication overall.

**TABLE 1.** Baseline characteristics of DCD liver transplants stratified by perfusion strategy

Variable	In-transit NMP	Back-to-base NMP	*P*
Recipient characteristics			
Recipient age, y	59.00 (51.00–65.00)	58.00 (51.00–65.00)	0.503
Recipient sex			0.354
Female	330 (35.3)	90 (32.0)	
Male	606 (64.7)	191 (68.0)	
Recipient ethnicity			0.064
White	673 (71.9)	219 (77.9)	
Black	36 (3.8)	12 (4.3)	
Hispanic	177 (18.9)	44 (15.7)	
Other	50 (5.3)	6 (2.1)	
Recipient BMI, kg/m^2^	28.36 (24.87–32.64)	28.89 (25.31–32.58)	0.306
MELD at transplant	19.00 (13.00–24.00)	19.00 (13.00–24.00)	0.927
Admission status			**0.045**
ICU	38 (4.1)	3 (1.1)	
Hospitalized, non-ICU	114 (12.2)	32 (11.4)	
Not hospitalized	784 (83.8)	246 (87.5)	
Waitlist time, d	75.50 (18.00–231.25)	85.00 (18.00–229.00)	0.988
Primary diagnosis			**0.028**
Noncholestatic cirrhosis	587 (62.7)	196 (69.8)	
Cholestatic cirrhosis	51 (5.4)	8 (2.8)	
Acute hepatic necrosis	4 (0.4)	0 (0.0)	
Metabolic disease	27 (2.9)	5 (1.8)	
Malignant neoplasm	236 (25.2)	56 (19.9)	
Other	31 (3.3)	16 (5.7)	
Prior abdominal surgery			0.239
Yes	500 (53.4)	148 (52.7)	
No	427 (45.6)	133 (47.3)	
Unknown	9 (1.0)	0 (0.0)	
Portal vein thrombosis			0.615
Yes	146 (15.6)	48 (17.1)	
No	790 (84.4)	233 (82.9)	
On dialysis			**0.044**
Yes	29 (3.1)	2 (0.7)	
No	907 (96.9)	279 (99.3)	
Donor characteristics			
Donor age (y)	46.00 (34.00–56.00)	46.00 (33.00–55.00)	0.353
Donor sex			0.852
Female	312 (33.3)	96 (34.2)	
Male	624 (66.7)	185 (65.8)	
Donor BMI, kg/m^2^	27.92 (24.21–32.55)	27.34 (23.16–31.72)	0.152
Donor diabetes			0.766
Yes	143 (15.3)	42 (14.9)	
No	786 (84.0)	238 (84.7)	
Unknown	7 (0.7)	1 (0.4)	
Heavy alcohol use			0.972
Yes	201 (21.5)	60 (21.4)	
No	698 (74.6)	209 (74.4)	
Unknown	37 (4.0)	12 (4.3)	
Donor cause of death			0.219
Anoxia	497 (53.1)	144 (51.2)	
Stroke	195 (20.8)	67 (23.8)	
Head trauma	189 (20.2)	62 (22.1)	
CNS tumor	5 (0.5)	0 (0.0)	
Other	50 (5.3)	8 (2.8)	
HCV NAT positive			1.000
Negative	911 (97.3)	274 (97.5)	
Positive	25 (2.7)	7 (2.5)	
LDRI	2.60 (2.27–3.00)	2.65 (2.30–3.09)	0.143
Transplant characteristics			
Preservation time, h	14.11 (10.45–18.28)	15.95 (13.70–18.37)	**<0.001**
Distance from donor hospital to transplant center (nautical miles)	115.50 (29.00–301.25)	102.00 (28.00–215.00)	0.063

Values are presented as median (IQR) for continuous variables and n (%) for categorical variables. *P* values calculated using the Wilcoxon rank-sum test or chi-square test, as appropriate. Bolded values indicate statistically significant values.

BMI, body mass index; CNS, central nervous system; DCD, donation after circulatory death; HCV, hepatitis C virus; ICU, intensive care unit; IQR, interquartile range; LDRI, liver donor risk index; MELD, Model for End-Stage Liver Disease; NAT, nucleic acid testing; NMP, normothermic machine perfusion.

Donor age, sex, BMI, diabetes status, and cause of death were comparable between groups. The median preservation time was longer in the back-to-base cohort (15.95 versus 14.11 h, *P* < 0.001), consistent with cold storage preceding perfusion initiation. The distance between the donor hospital and the transplant center did not differ significantly between groups (102 versus 115.5 nautical miles, *P* = 0.063).

### Posttransplant Outcomes

**FIGURE 5.**
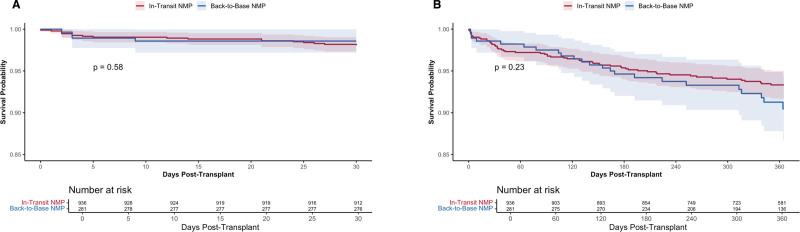
Kaplan-Meier analysis of composite graft failure stratified by perfusion strategy after DCD liver transplantation. Thirty-day (A) and 1-y (B) survival probability curves comparing in-transit NMP vs back-to-base NMP. Composite graft failure was defined as death or retransplantation. Shaded regions indicate 95% confidence intervals. *P* values derived from log-rank tests. DCD, donation after circulatory death; NMP, normothermic machine perfusion.

Median length of stay was significantly shorter in the in-transit NMP group compared with the back-to-base group (8 versus 9 d, *P* < 0.001). Acute rejection before discharge occurred in 3.3% of in-transit NMP recipients and 5.3% of back-to-base recipients, although this difference was not statistically significant (*P* = 0.15). At 30 d posttransplant, survival was 98.1% for in-transit NMP and 98.6% for back-to-base NMP (log-rank *P* = 0.58; Figure 5). At 1 y, survival was 93.3% and 90.5%, respectively (*P* = 0.23).

To guide covariate selection and reduce overfitting in the adjusted model, we first applied elastic net penalized Cox regression, followed by least absolute shrinkage and selection operator regression as a sensitivity analysis. Both methods consistently selected 3 predictors with nonzero coefficients: preservation strategy, dialysis at transplant, and hospitalization status at transplant. These were included in the final traditional Cox proportional hazards model. In this model, back-to-base NMP had a higher, but not statistically significant, hazard of 1-y graft failure compared with in-transit perfusion (hazard ratio [HR] 1.46 [95% confidence interval (CI), 0.90-2.38]; *P* = 0.13). Patients not hospitalized at the time of transplant had a lower hazard of graft failure than those in the ICU (HR 0.38 [95% CI, 0.15-0.99]; *P* = 0.049). Dialysis and non-ICU hospitalization were not significantly associated with 1-y graft failure (Table 2).

**TABLE 2.** Multivariable Cox regression results for 1-y graft failure

Variable	Adjusted HR	95% CI	*P*
Back-to-base NMP (vs in-transit NMP)	1.46	0.90-2.38	0.127
Dialysis at transplant	1.50	0.49-4.56	0.480
Hospitalized, non-ICU (vs ICU)	0.58	0.21-1.62	0.296
Not hospitalized (vs ICU)	0.38	0.15-0.99	**0.049**

Reference category is in-ICU at time of transplant. Bolded value indicates statistically significant value.

CI, confidence interval; HR, hazard ratio; ICU, intensive care unit; NMP, normothermic machine perfusion.

### Sensitivity Analysis Among Grafts With CIT ≥10 h

To assess whether differences in preservation time biased outcomes, we performed a sensitivity analysis restricted to recipients in both cohorts with CIT ≥10 h (n = 1001). Baseline characteristics remained largely similar between groups, including recipient age, MELD score, and donor demographics. Median CIT was comparable (15.82 versus 15.95 h, *P* = 0.85). In-transit recipients were more often White and had a longer donor distance (125 versus 102 miles, *P* = 0.006).

One-year graft survival remained statistically similar between groups (93.3% in in-transit versus 91.8% in back-to-base, log-rank *P* = 0.23). In a Cox proportional hazards model adjusting for dialysis and recipient medical condition at transplant, back-to-base perfusion was not associated with a significant difference in graft failure risk compared with in-transit (HR 1.34 [95% CI, 0.81-2.21], *P* = 0.26).

### Waitlist Duration Analysis

In a center-adjusted mixed-effects model, back-to-base NMP was associated with a longer waitlist time compared with in-transit NMP (ratio of geometric mean wait days = 1.30; 95% CI, 1.02-1.65; *P* = 0.03). Adjusted mean wait times were 86 d (95% CI, 60-123) for back-to-base and 66 d (95% CI, 49-90) for in-transit recipients.

## DISCUSSION

In this national analysis of DCD liver transplants after NMP, we found that back-to-base and in-transit strategies were implemented in distinct clinical and geographic contexts but yielded similar short- and intermediate-term graft outcomes. Back-to-base NMP was more common in the Midwest and Southeast and involved longer preservation times, whereas in-transit NMP was more frequently used in the Western United States and among recipients with higher clinical acuity, including those on dialysis. Notably, in-transit NMP was associated with a significantly shorter hospital length of stay. Despite these contextual and logistical differences, rates of early rejection and 1-y graft survival were comparable. These findings suggest that both NMP delivery strategies are viable and can be tailored to institutional preferences and infrastructure.

As the first national study to compare clinical outcomes between in-transit and back-to-base NMP strategies, we found that both approaches yielded comparable short- and intermediate-term graft survival among DCD liver transplant recipients. Both NMP platforms have been investigated in prospective trials.^18-21^ The OrganOx Metra device has been evaluated in the multicenter VITTAL trial, which demonstrated that 71% of initially discarded livers met viability criteria and achieved 100% 90-d graft survival. A subsequent randomized trial by Nasralla et al^19^ found that NMP reduced graft injury, improved preservation time, and lowered discard rates compared with SCS. Most recently, the US-based trial led by Chapman et al^20^ demonstrated that back-to-base NMP using the OrganOx system had comparable early allograft dysfunction (EAD) rates, with higher-risk liver benefiting more from the device. In contrast, the TransMedics OCS device was assessed in the PROTECT trial, which reported lower rates of EAD and ischemic biliary complications compared with cold storage. These trials confirm the safety and efficacy of each platform, but direct comparisons have been lacking.

Our study complements this evidence by showing that, in a national cohort of DCD recipients, both strategies yield similar early rejection rates and survival outcomes. Notably, livers perfused via a back-to-base approach experienced significantly longer preservation times (16.0 versus 14.1 h), but both approaches appear well suited to support the prolonged preservation time necessary in modern transplant logistics. Furthermore, because our classification strategy inherently excluded short CIT cases from the back-to-base group, we performed a sensitivity analysis limited to cases with CIT ≥10 h across both groups. This demonstrated no significant difference in overall CIT and yielded similar graft survival, supporting the validity of our overall findings. In-transit recipients had a significantly shorter hospital length of stay (8 versus 9 d), suggesting potential advantages in postoperative recovery. Although we did not include a SCS comparator, prior institutional work from our group reported a median length of stay of 13 d among SCS recipients,^6^ indicating that both NMP strategies offer improved perioperative efficiency compared with conventional preservation.

We observed substantial geographic and center-level variation in the adoption of NMP strategies. Back-to-base perfusion was most common in the Midwest, particularly UNOS Region 10, where Cleveland Clinic—one of the highest-volume transplant centers in the United States—has been a leading adopter of back-to-base NMP. Their institutional series,^8^ which included their Abu Dhabi site, demonstrated that back-to-base NMP is feasible for high-risk grafts, with outcomes comparable with SCS and cost neutrality when both organ acquisition and hospitalization expenses are considered. In contrast, in-transit NMP was more frequently used in the Western United States, especially UNOS Region 5. This pattern may reflect the influence of Mayo Clinic Arizona, where Nguyen et al^7^ reported their center’s experience with 279 DCD-NMP liver transplants between 2019 and 2023, with lower rates of EAD, reduced transfusion needs, and shorter hospital stays relative to both SCS and donation after brain death recipients. These regional patterns likely reflect more than clinical preference alone. Platform selection is shaped by logistical constraints, institutional infrastructure, and center expertise. Back-to-base NMP offers simplified logistics by avoiding the need to transport devices and personnel, whereas in-transit systems reduce overall exposure to cold ischemia. Notably, the majority of NMP transplants were performed by a small subset of centers (Figure 4), highlighting the potential impact of startup costs, specialized training, and reliance on experienced procurement teams in limiting broader adoption.

Cost considerations may further drive institutional preferences. Organ acquisition costs (OACs)—which include recovery, perfusion disposables, transport, and personnel—differ substantially across platforms. In a back-to-base model using the OrganOx Metra, Wehrle et al^8^ reported a median OAC of $88 923 for DCD-NMP cases. In contrast, our group’s single-center analysis found significantly higher OAC in TransMedics OCS cases ($135 930 versus $50 940 for SCS, *P* < 0.001), driven primarily by $83 900 in supply and personnel charges associated with the device.^6^ OPO and transport costs were relatively comparable. Importantly, the transition from SCS to NMP corresponded with a steep rise in median OAC—from $51 051 in 2021 (pre-NMP) to $135 110 in 2023 (predominantly NMP;  *P* < 0.001). These financial differences may influence not only platform selection but also whether NMP is adopted at all. Although both systems improve logistical flexibility and support marginal graft utilization, their differing cost structures may impact access, particularly for lower-volume centers or those with limited institutional resources.

We also observed modest differences in recipient acuity between platforms. In-transit NMP recipients were more likely to be on dialysis at transplant (3.1% versus 0.7%) and had a slightly higher proportion of ICU admissions, which may reflect more aggressive use in higher-acuity patients or broader recipient inclusion at experienced centers. Meanwhile, back-to-base recipients were more frequently not hospitalized at the time of transplant, possibly reflecting a preference for elective operating room scheduling. However, these differences were small and did not translate into differences in rejection or 1-y survival. Together, these findings reinforce the idea that logistical and institutional factors—not recipient clinical status—are likely the dominant drivers of platform selection. Further research is needed to clarify how patient, donor, and system-level factors interact to shape outcomes and inform optimal deployment of NMP.

Beyond posttransplant outcomes, the impact of preservation strategy on waitlist dynamics is an important consideration. Prior 1- and 2-center studies have shown that programmatic adoption of back-to-base NMP was associated with marked reductions in waitlist time and mortality by facilitating the use of marginal grafts and enabling access for lower-MELD candidates.^22,23^ In contrast, in our study, the adjusted mean waitlist time was 86 d for back-to-base NMP and 66 d for in-transit NMP after controlling for age, sex, MELD score, diagnosis, and center-level effects. These findings likely reflect operational and geographic factors rather than intrinsic differences in platform performance. In-transit NMP allows graft perfusion to begin at the donor hospital, potentially expediting procurement and allocation across regions, whereas back-to-base programs require transport of the organ to the transplant center before initiating perfusion. Collectively, these results suggest that, at the national level, access and logistics—not device efficacy—are the principal drivers of observed differences in waitlist duration between NMP strategies.

This study has several limitations. Most notably, perfusion strategy was inferred on the basis of graft preservation time rather than directly reported, which introduces the potential for exposure misclassification. However, this is not the first time assumptions have been necessary in national registry studies. Zhou et al^24^ recently used similar inference methods to distinguish static versus portable ex vivo lung perfusion strategies in lung transplantation, and nearly all SRTR-based normothermic regional perfusion studies to date have relied on surrogate markers such as donor death and cross-clamp time to infer recovery technique.^25-28^ That said, our classification thresholds were grounded in clear bimodal histograms, corroborated by recently published correspondence,^10,11^ and supported by informal conversations with SRTR stakeholders who confirmed that initiation of machine perfusion is recorded at the donor hospital. The geographic patterns in our data also further support our classification, with UNOS Region 10 (home to Cleveland Clinic) leading in back-to-base use, whereas Region 5 (likely driven by Mayo Clinic Arizona) led in in-transit use. Still, we acknowledge that a small minority of OrganOx cases may have been perfused at the donor hospital rather than at the transplant center, potentially resulting in some overlap across groups. Additionally, although we excluded livers undergoing hypothermic machine perfusion, we recognize that the ongoing PILOT trial included delayed end-ischemic perfusion strategies that could resemble back-to-base NMP.^29^ However, perfusion times were short—median cold preservation durations of 6.4–6.8 h and a pre-perfusion CIT of 4.1 h—well below our 10-h threshold, making inclusion of these grafts unlikely.

In addition, the SRTR data set has inherent structural limitations that warrant emphasis. CIT is reported as the total duration from donor cross-clamp to recipient anastomosis, without distinguishing between cold storage and machine perfusion phases. As a result, back-to-base cases appear to have prolonged “cold” times although much of this period occurs under normothermic conditions. This limitation precludes accurate comparison of preservation durations between strategies and represents a major gap in the SRTR schema. Similarly, SRTR does not reliably capture critical posttransplant complications, such as ischemic cholangiopathy and nonanastomotic biliary strictures—outcomes central to evaluating the safety and efficacy of DCD and machine-perfused grafts. Recent work proposing standardized Core Outcome Sets for liver transplantation highlights the need to include biliary, graft, and long-term morbidity endpoints to enable consistent benchmarking across perfusion approaches.^30,31^ Incorporating such metrics, along with explicit device-level and timing data, will be essential for modernizing the SRTR and improving the precision of national perfusion analyses. Finally, although this is the first national comparison of in-transit versus back-to-base NMP, our follow-up is limited to 1 y, and, as with any retrospective study, the potential for residual confounding from unmeasured variables remains.

## CONCLUSIONS

In this national analysis of NMP utilization and outcomes in DCD liver transplantation, we found that back-to-base and in-transit strategies offer comparable early- and intermediate-term outcomes despite being used in distinct geographic contexts. The observed variation in platform adoption likely reflects underlying logistical demands, institutional expertise, and cost structures rather than differences in clinical efficacy. Our findings suggest that both approaches can be integrated into routine practice without compromising patient outcomes, offering centers the flexibility to tailor preservation strategies to local workflows and resource availability. Moving forward, efforts to expand NMP adoption should prioritize improving national data infrastructure to enable direct platform identification, refining reimbursement frameworks that account for true OACs, and facilitating broader access to machine perfusion technology, particularly for lower-volume centers. As the field moves toward standardizing graft assessment and optimizing logistics, both NMP strategies appear well positioned to support further expansion of the donor pool and reduce the burden of liver graft discard in the US system.
